# Abdominal microbial communities in ants depend on colony membership rather than caste and are linked to colony productivity

**DOI:** 10.1002/ece3.5801

**Published:** 2019-11-14

**Authors:** Francisca H. I. D. Segers, Martin Kaltenpoth, Susanne Foitzik

**Affiliations:** ^1^ LOEWE Centre for Translational Biodiversity Genomics (LOEWE‐TBG) Frankfurt Germany; ^2^ Behavioural Ecology and Social Evolution Institute of Organismic and Molecular Evolution Johannes Gutenberg University Mainz Germany; ^3^ Evolutionary Ecology Institute of Organismic and Molecular Evolution Johannes Gutenberg University Mainz Germany; ^4^ Present address: Applied Bioinformatics Group Institute of Cell Biology & Neuroscience Goethe University Frankfurt Germany

**Keywords:** 16S rRNA sequencing, colony fitness, colony phenotype, gut bacteria, social insects, *Temnothorax*

## Abstract

Gut bacteria aid their host in digestion and pathogen defense, and bacterial communities that differ in diversity or composition may vary in their ability to do so. Typically, the gut microbiomes of animals living in social groups converge as members share a nest environment and frequently interact. Social insect colonies, however, consist of individuals that differ in age, physiology, and behavior, traits that could affect gut communities or that expose the host to different bacteria, potentially leading to variation in the gut microbiome within colonies. Here we asked whether bacterial communities in the abdomen of *Temnothorax nylanderi* ants, composed largely of the gut microbiome, differ between different reproductive and behavioral castes. We compared microbiomes of queens, newly eclosed workers, brood carers, and foragers by high‐throughput 16S rRNA sequencing. Additionally, we sampled individuals from the same colonies twice, in the field and after 2 months of laboratory housing. To disentangle the effects of laboratory environment and season on microbial communities, additional colonies were collected at the same location after 2 months. There were no large differences between ant castes, although queens harbored more diverse microbial communities than workers. Instead, we found effects of colony, environment, and season on the abdominal microbiome. Interestingly, colonies with more diverse communities had produced more brood. Moreover, the queens' microbiome composition was linked to egg production. Although long‐term coevolution between social insects and gut bacteria has been repeatedly evidenced, our study is the first to find associations between abdominal microbiome characteristics and colony productivity in social insects.

## Graphical abstract

In this study, we assessed the effects of environment, colony, and individual differences on abdominal bacterial communities in the ant *Temnothorax nylanderi*. Most interestingly, we discovered colony‐specific abdominal microbiomes in this ant, which suggests a role of host genetics, the social environment, or both in shaping the abdominal community. On top of that, we observed for the first time that colony microbial community diversity is positively correlated with productivity in a social insect.

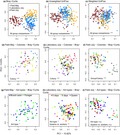

## 1 INTRODUCTION

Animals contain bacterial communities within their guts that are generally thought to be a fundamental component of their bodies (Sommer & Bäckhed, 2013). These bacteria carry genes with enzymatic functions that the animal hosts would otherwise not have access to (Engel & Moran, 2013; Zilber‐Rosenberg & Rosenberg, 2008). A high diversity in gut bacterial communities exists between species, which are often tightly linked to an animal's lifestyle, especially its diet (Anderson et al., 2012; Ley et al., 2008; Rubin, Kautz, Wray, & Moreau, 2019). For instance, wood‐feeding termites harbor bacteria in their gut, which produce enzymes that aid in the digestion of their high‐cellulose food (Brune, 2014). Yet, within animal species, the composition of gut bacterial communities can vary from individual to individual and can influence the phenotype of the host. For example, gut bacteria can affect an animal's nutritional state (Turnbaugh, Bäckhed, Fulton, & Gordon, 2008), smell (Li et al., 2013), and ability to resist pathogens (Koch & Schmid‐Hempel, 2011). In turn, a host's genotype (e.g., Davenport, 2016; Näpflin & Schmid‐Hempel, 2018; Zomer et al., 2009) and diet (e.g., Carmody et al., 2015; D'Alvise et al., 2018; Larson et al., 2014) can determine the composition of its gut bacterial community.

It is in the interest of the host to maintain a beneficial microbial community, as the presence or absence of certain bacteria in the gut can have severe fitness consequences. A host can contribute to the maintenance of an advantageous gut microbiome by favoring some bacterial strains over others through physiological or immunological adaptations (e.g., Bution & Caetano, 2008; Lanan, Rodrigues, Agellon, Jansma, & Wheeler, 2016) and by the evolution of inheritance mechanisms that ensure the selective transfer of fitness‐enhancing strains from one generation to the other (Engel & Moran, 2013; Salem, Flórez, Gerardo, & Kaltenpoth, 2015). In addition, the environment plays an important role for the inoculation and replenishment of gut symbionts in many animal species (e.g., Blum, Fischer, Miles, & Handelsman, 2013; Kikuchi, Hosokawa, & Fukatsu, 2007). Social living promotes the exchange of gut bacteria among group members through a shared environment and frequent physical interactions (Archie & Tung, 2015; Engel & Moran, 2013; Onchuru, Martinez, Ingham, & Kaltenpoth, 2018). Resultantly, correlations between the gut community compositions of nestmates have been found in insects (Koch & Schmid‐Hempel, 2011), birds (White et al., 2010), and mammals (Leclaire, Nielsen, & Drea, 2014).

Social insect societies are highly integrated biological systems, containing multiple generations, which function as if they are one “superorganism” (Seeley, 1989; Hölldobler & Wilson, 1990). For instance, social insects are often considered to have “social stomachs”: Specialist foraging workers bring food back to the nest, which is subsequently processed and distributed among the other workers, larvae, and reproductives (e.g., Dussutour & Simpson, 2009; Nixon & Ribbands, 1952). Physical interactions with the purpose of communicating and food sharing are frequent among colony members of many social insects. One such interaction is mouth‐to‐mouth or anus‐to‐mouth contact called “trophallaxis” in which one nestmate transfers a liquid to another (e.g., Grüter, Acosta, & Farina, 2006; Hamilton, Lejeune, & Rosengaus, 2011). These kinds of social exchanges provide opportunities for bacteria to jump hosts. Also, bacterial transmission via nest material (e.g., brood comb) has been demonstrated to promote the spread of microbes through social insect colonies (Powell, Martinson, Urban‐Mead, & Moran, 2014). A guaranteed transfer of bacteria between group members over several generations facilitates the coevolution between gut bacteria and animal species (Engel & Moran, 2013; Lombardo, 2008). Accordingly, phylogenetic analyses of different social insect species and their gut symbionts are suggestive of long periods of coevolution (Anderson et al., 2012; Hongoh et al., 2005; Kwong et al., 2017; Martinson et al., 2011; Russell et al., 2009).

In Hymenopteran societies (ants, bees, wasps) with a sole singly‐mated queen, workers are related by 0.75 due to their haplodiploid sex determination system, while queens are related by 0.5 to their daughters, the workers. Despite this high relatedness, colonies often consist of individuals widely differing in physiology and behavior. In eusocial Hymenoptera, the queen specializes in egg laying. She is often the oldest and largest individual in the colony and has well‐developed ovaries compared with her daughters, the workers. Therefore, a queen's physiology is likely to represent a different niche to colonizing bacteria. Also, it is conceivable that queens have different needs with regard to nutrition than workers and thus may require different nutritional symbionts. However, even within the worker caste, individuals can differ in their physiology, as workers that perform brood care duties are often the youngest, and differ hormonally from the older workers, which take over nest defense or foraging (Kohlmeier, Alleman, Libbrecht, Foitzik, & Feldmeyer, 2019; Robinson, 1992). On top of that, outside workers, such as foragers, are more likely to come in contact with foreign bacterial strains than, for example, brood carers whose duties restrict them to the inside of the nest. Thus, even though the members of a social insect colony share the same nest environment, interact frequently, and are often highly related, we can expect variation in gut microbial communities within a colony that may correlate with caste and/or the tasks that an individual performs.

In line with this, Poulsen et al. (2014) noted that the gut microbiome characteristics of fungus‐growing termites correspond to their role in the division of labor. More specifically, workers harbor more complex gut communities than queens, presumably more suited to process food, while queens are supplied with fungal material by the workers. Reproductive caste‐specific microbiomes have also been found in the honey bee (*Apis mellifera*): The gut communities of queens are dominated by different taxonomic groups than those of workers, with queens harboring a much higher abundance of alphaproteobacterial strains (Kapheim et al., 2015; Tarpy, Mattila, & Newton, 2015). Yet, it remains unknown whether these differences are functionally relevant.

Also within the worker caste, differences in gut bacterial communities have been found. Age‐matched honey bees that perform tasks inside the hive, such as brood care, had more diverse gut communities than foragers (Jones et al., 2018). Within worker caste, differences in gut communities seem to be especially pronounced between newly eclosed and older social insect workers: At eclosion from the pupal stage, honey bee workers are generally devoid of gut bacteria (Martinson, Moy, & Moran, 2012), but through frequent social interactions with older bees and exposure to hive materials young workers acquire different species of symbionts within several days after emergence (Powell et al., 2014). Likewise, newly eclosed individuals (callows) and late stage pupae of cephalotine (Russell et al., 2009) and attine ants (Zhukova, Sapountzis, Schiøtt, & Boomsma, 2017), respectively, lack bacteria in their guts. Holometabolous insects, such as the Hymenoptera, shed the entire inner larval gut epithelium including their gut content during metamorphosis (Hakim, Baldwin, & Smagghe, 2010), which can lead to the clearance of symbionts from the gut. However, in some holometabolous insects, gut symbionts persist through metamorphosis due to actions of the host, the symbiont, or both (e.g., Johnston & Rolff, 2015). This might explain why in some attine ants late stage pupae do not differ from older workers in either absolute abundance of bacteria or in gut community diversity (Zhukova et al., 2017). Thus, the development of the gut bacterial communities throughout the life of social insect workers likely varies depending on their biology.

Here we examined the gut bacterial community of the common small European ant, *Temnothorax nylanderi* (Foerster, 1850), by high‐throughput 16S rRNA sequencing of the abdomens of individual ants. We were mainly interested in whether reproductive and worker castes (callows, brood carers, foragers, and queens) harbor distinct microbiota, as we would expect due to the physiological differences between these ant types and due to the small scale environmental differences to which they are exposed. We sampled two types of callow workers, 0 and 5 days old, to investigate whether newly emerged *T. nylanderi* workers have a reduced gut community diversity as has been found for some other social insects (e.g., Martinson et al., 2012; Zhukova et al., 2017). By sampling multiple workers from many colonies (21), we were also able to determine whether *T. nylanderi* colonies have characteristic abdominal microbiomes. As a first step toward examining whether abdominal communities potentially affect the reproductive success of a *T. nylanderi* ant colony, we tested for correlations between colony fitness traits (e.g., per capita brood production, number of eggs in the nest, and worker number) and community characteristics. Lastly, we tested how consistent abdominal communities in *T. nylanderi* are in response to environmental influences, as little is known about differentiation in gut communities over time in the field and after transfer to laboratory conditions. Therefore, we repeatedly sampled individuals from the same colonies directly after collection in the field and after 2 months of laboratory housing. When we sampled ants in the laboratory, we again collected colonies from the field, to disentangle the effects of environment and season on the abdominal microbiome. Our expectation was that colonies transferred to the laboratory would become more similar to each other in their community composition, because all colonies were exposed to the same homogeneous laboratory environment.

## 2 MATERIALS AND METHODS

### 2.1 Study species and sample collection

**Figure 1:**
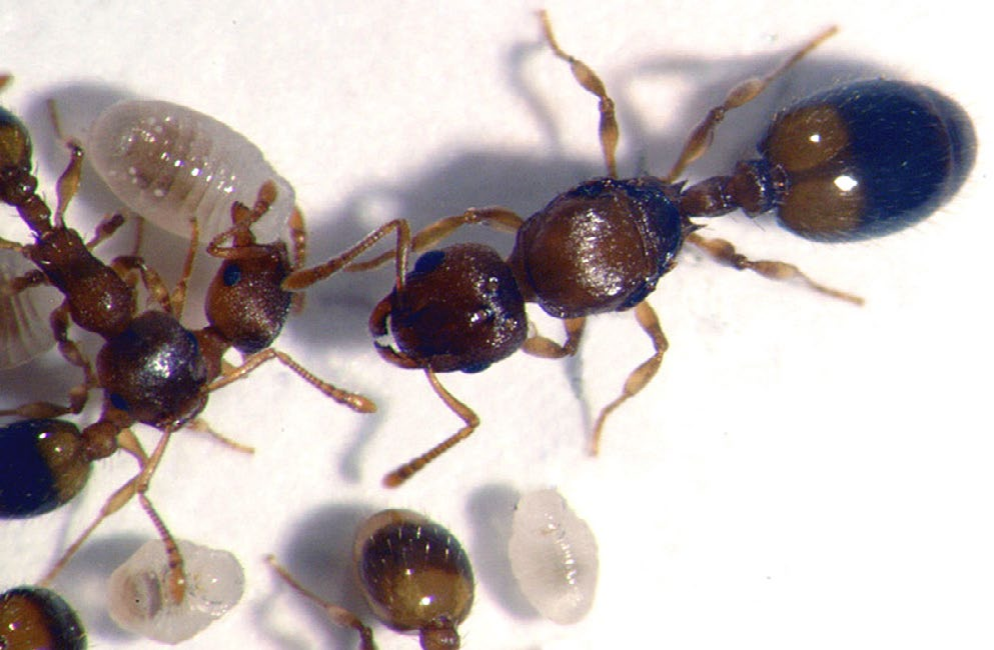
A *Temnothorax nylanderi* queen (largest individual) with workers and larvae

*Temnothorax nylanderi* is a small myrmicine ant (Figure 1) that is found in forests throughout Europe. Its colonies are composed of several dozen to a few hundred individuals, and they inhabit pre‐existing cavities in wood (e.g., broken‐off branches, pine cones, and acorns) on the forest floor (Foitzik & Heinze, 1998). *Temnothorax* ants are thought to be trophic generalists, and their diet includes dead insects, honey dew, vertebrate feces, and seeds (reviewed in Prebus, 2017).

**Figure 2:**
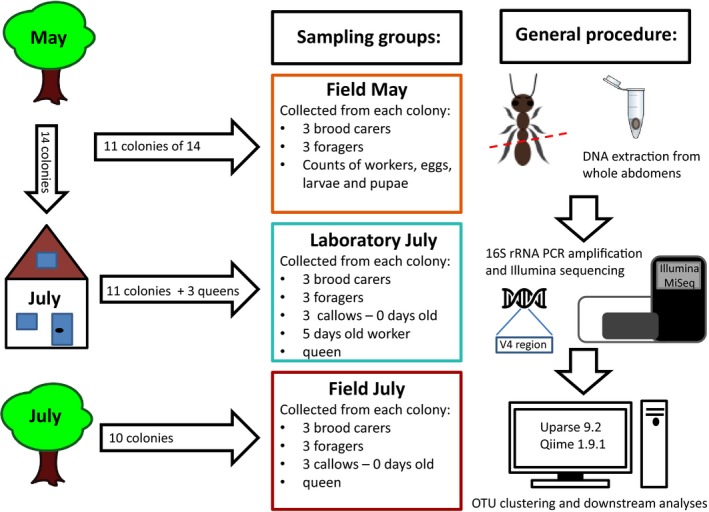
An overview of the sampling procedure and subsequent high‐throughput sequencing of the 16S rRNA gene. In May, fourteen *Temnothorax* *nylanderi* colonies were collected from the forest and kept in the laboratory for 2 months. Although 14 colonies were initially collected from the forest in May, from only 11 colonies we have worker samples for the Field May and Laboratory July sampling group, due to loss of samples from three colonies. In July, 10 new colonies were collected from the same field site

In May 2016, 14 *T. nylanderi* colonies were collected in the Chausseehaus Forest in Wiesbaden, Germany. All colonies were residing in branches, which were broken open to reveal the nest. Subsequently, the sticks were stored in plastic bags and transported to our laboratory. Overnight, the colonies were offered artificial nests made from a piece of holed out Plexiglas sandwiched by two glass microscopy slides. All colonies moved into their new artificial housing by morning, and the nests were placed into plastic boxes with plaster floors. The ants had access to sterilized water but were not fed. We counted for each colony the workers, eggs, larvae, and pupae. After 2 days of settling, we collected three brood carers and three foragers from 11 of the 14 colonies (samples were lost from three colonies; Figure 2). We assumed that individuals close to the brood were brood carers and those outside the nest were foragers. Assignment to worker caste based on a single observation is highly reliable in *Temnothorax* ants, including our focal species *T. nylanderi* (Beros, Enders, Menzel, & Foitzik, 2019; Kohlmeier, Feldmeyer, & Foitzik, 2018; Kohlmeier et al., 2017). The sampled workers were stored in clean 1.7 ml Eppendorf tubes at −20°C until DNA extraction.

In the laboratory, ant colonies were kept inside their plastic boxes in a climate chamber at simulated spring and summer conditions (12:12 hr, 20/12°C light:dark and 14:10 hr, 25/20°C light:dark, respectively). Twice a week, the ants were provided ad libitum with honey, crickets, and water. After 2 months, we sampled again three brood carers and three foragers from the same 11 colonies as before, according to the same criteria, to assess whether and how the abdominal bacterial community changes under the influence of laboratory conditions. In addition, we collected three recently eclosed workers (callows) that can be recognized by their light cuticle. Another three callows were marked with thin metal wires and recaptured after 5 days. From all 14 colonies, we sampled the queen (Figure 2).

During the same time period as when we sampled ants from the laboratory colonies (early July 2016), we collected ten *T. nylanderi* colonies from the same forest. After returning to the laboratory, we directly sampled the queen and three callow workers per colony. We followed the same procedure as described above to additionally collect three brood carers and foragers from each colony (Figure 2). These samples provided a control for time and seasonal effects on the ant abdominal communities. Again the ants were kept in clean tubes at −20°C until DNA extraction.

### 2.2 DNA extraction

After rinsing the ant in 0.1% SDS and distilled water, we separated the abdominal segments II–VII from the thorax with clean dissection tools. This included the gaster, petiole, and postpetiole. On the day of DNA extraction, the abdomens were homogenized by submersing them in liquid nitrogen and subsequently crushing them with plastic mortars. The homogenized abdomens of each ant represented one sample. By using the abdomens instead of the guts, we prevent the loss of bacteria during dissection; however, bacteria living in and on other *T. nylanderi* tissues, such as the ovaries, might have been inadvertently sampled. We assume, though, that the bacteria our analysis detected are predominantly gut colonizers.

DNA from each entire abdomen was extracted with a commercially available kit (MasterPure™, from EpiCentre). We followed the manufacturer's instructions and added an extra lysozyme step to the protocol to ensure the lysis of Gram‐positive bacterial cell walls: After adding 2 μl of ready‐to‐lyse lysozyme (250 U/µl TES buffer) to each sample, the samples were incubated for 30 min. at 37°C. After DNA extraction, samples were stored at −80°C until sequencing. As controls for external bacterial contamination, we extracted DNA from (a) the solutions used for washing the ants, (b) rinse off from dissection tools, (c) swaps of the plaster nests, and (d) the sustenance provided to the laboratory colonies (tap water, honey, and cricket leg).

### 2.3 Sequencing

After DNA extraction, 16S rRNA amplicon sequencing was performed by StarSEQ GmbH: The V4 region was amplified using the 515f‐806rB primer pair, followed by Illumina MiSeq sequencing, generating 2 × 300 nt paired‐end reads (overlapping). Demultiplexing, adapter trimming, and quality filtering were done according to the in‐house Illumina MiSeq workflow by StarSEQ. This resulted in 10,579,030 reads, of which 97.3% had a mean phred score higher than 25.

### 2.4 Processing of microbiota sequencing data

Operational taxonomic units (OTUs) were found by mainly following the Uparse 9.2 (Edgar, 2013) pipeline (http://www.drive5.com/uparse). The merging of the paired‐end reads was only marginally successful (on average 65.6% ± 5.4 *SD* successfully merged per sample). Therefore, we continued the pipeline using only the forward reads. The reads were filtered with Usearch 9.2. (maxEE cutoff set to 1). The filtered reads were dereplicated with the “‐fastx_uniques” step before we clustered the OTUs with “‐cluster_otus,” which at the same time removes chimeras. Singleton unique sequences were excluded from OTU clustering by setting “‐minsize” to 2. To generate an OTU table, we mapped the unfiltered forward reads to the OTUs with the usearch_global algorithm using a 97% identity cutoff (on average, 94.2% ± 3.1 *SD* reads were assigned to an OTU per sample). With the Qiime 1.9.1 script “assign_taxonomy.py” (Caporaso et al., 2010), we allocated taxonomic groups to the OTUs found by Usearch 9.2., using the Greengenes database gg_13_8. Subsequently, we deleted OTUs classified as chloroplasts or mitochondria, but archaeal OTUs were kept. Sixty‐seven OTUs were unassigned, and as BLAST searches indicated that they were of fungal origin, they were discarded. Also, OTUs were removed from the table if they were not, in terms of relative abundance, at least five times more common in an experimental sample than in a control and we only kept OTUs that contributed at least 0.1% to the community of at least one sample. Our decontamination protocol was less conservative than other recent studies (e.g., Hu et al., 2017; Łukasik et al., 2017) because we wanted to avoid the removal of false‐positive contaminants. The OTU table we used for our analyses could still contain some bacterial strains that were present in, for example, our DNA extraction kit. However, all samples were extracted and sequenced simultaneously, therefore ruling out any systematic contamination correlating with sampling groups, colonies, and ant types. Thus, we are convinced that any differences we find in abdominal communities among our samples represent real biological differences. We provide the OTU table before and after filtration in the Tables S1a and S1b.

The inclusion of cricket material as contaminant controls could potentially lead to the faulty exclusion of bacterial strains that are colonizers of both ant and cricket tissue. Therefore, we did a closer examination of the OTUs that were deleted from the OTU table due to the inclusion of the cricket material: The OTUs in question did not have high percentile abundances in the ant samples and were mainly human‐associated and environmental bacteria (Table S2). Thus, we concluded that the use of the cricket material likely did not result in the wrongful exclusion of OTUs that could be important *T. nylanderi* symbionts.

After the removal of likely contaminants, we were left with 647 OTUs, including one assigned to the endosymbiotic genus *Wolbachia*. Although *Wolbachia* can colonize guts (Andersen, Boye, Nash, & Boomsma, 2012), by extracting DNA from the abdomen, also DNA from *Wolbachia* present in the ovaries and other abdominal tissues was sequenced and therefore we excluded it from our analysis. We constructed a phylogenetic tree with the representative sequences for all OTUs as determined by the ‐cluster_otus step of the Uparse 9.2 pipeline. For this, we aligned the sequences with pynast and we subsequently computed the phylogeny with the FastTree method as implemented in Qiime 1.9.1., using the default settings.

After deleting sequences that were attributed to *Wolbachia*, the mean number of sequences of our samples was relatively low and quite variable among samples (on average 5,366 ± 4,757 *SD*, Table S3). To check whether our sequencing depth per sample was sufficient to reflect OTU diversity, while not having to exclude too many samples, we first performed multiple rarefaction in Qiime from 100 to up to 1,500 sequences with increments of 100 and a 100 iterations each time. For all ant types, the number of discovered OTUs shows a minor increase after a sequence number of about 800 per sample (Figure S1). Therefore, we decided to exclude samples with fewer than 800 sequences. This excluded eleven samples of which five were queen samples (mainly due to their high amount of *Wolbachia* reads), one forager sample, four brood carer samples, and one callow worker of 0 days old.

To further ensure that samples were not outliers due to low sequence numbers, we performed an ordination analysis with beta diversity distances and checked whether samples group together by the number of sequences. Before calculating several beta diversity indices (Bray–Curtis, weighted and unweighted UniFrac) in Qiime, we normalized the OTU table with the metagenomeSeq library (Paulson, Pop, & Bravo, 2013) from the Bioconductor project (Gentleman et al., 2004) in R 3.4.0 (R Development Core Team). Normalization instead of rarefying an OTU table is recommended by McMurdie and Holmes (2014). Based on the beta diversity matrices, principal coordinates were calculated with the principal_coordinates.py script in Qiime. These coordinates were plotted and the samples with the lowest numbers of sequences were colored (Figure S2). These plots showed no clustering of samples according to the number of sequences, suggesting that deleting samples with less than 800 sequences was enough to reduce noise due to sequencing depth.

### 2.5 Quantitative real‐time PCR analysis

To investigate whether Shannon's diversity is confounded by bacterial abundance, we used quantitative real‐time PCR (qrt‐PCR) on the Field May samples. Total bacterial 16S copy numbers per abdomen were estimated with universal 16S primers (Univ16SRT‐F: 5′‐ACTCCTACGGGAGGCAGCAGT‐3′; Univ16SRT‐R: 5′‐TATTACCGCGGCTGCTGGC‐3′; Clifford et al., 2012) on a Rotor‐Gene Q cycler (Qiagen) in final reaction volumes of 10 μl containing 0.5 μl of each primer (10 μM), 5 μl SYBR‐mix, 3 μl of qPCR H_2_O, and 1 μl of either template or standard or negative control (H_2_O). The following cycling conditions were used: 95°C denaturation for 10 s, 70–64°C touchdown annealing for the first six cycles, then 64°C annealing for the remaining 34 cycles for 15 s, extension of 72°C for 10 s, and a final melting curve analysis from 65°C to 99°C with a temperature increase of 1°C for each step. The DNA concentration of each sample was deducted from a standard curve made with a tenfold dilution series (10^4^–10^10^) of 16S DNA amplicons derived from a PCR with primers Univ16SRT‐F and Univ16SRT‐R and a combined DNA extract of the bacterial gut microbiota of *Dysdercus fasciatus*, *Pyrrhocoris apterus*, and *Probergrothius angolensis* (Hemiptera, Pyrrhocoridae) as template, which contains a diverse mixture of Gram‐positive and Gram‐negative bacteria. The PCR product was purified with the innuPREP PCRpure Kit (Analytik Jena), and the concentration of purified product was measured by Nanodrop (Thermo Scientific). Due to depletion of the samples by the Illumina MiSeq analysis, only one replicate was done for each sample. Two samples did not show any amplification, and one sample was an outlier by nearly tenfold compared to the second highest measured value and was thus excluded from the analysis.

### 2.6 Data analysis

We tested for significant effects of sampling group (Field May, Laboratory July, and Field July), colony identity, and ant type (0 days old, 5 days old, brood carers, foragers, and queens) on the beta diversity indexes using permutational multivariate analysis of variance (PERMANOVA) with 999 permutations. The PERMANOVA tests were done with the adonis function from the package vegan (Oksanen et al., 2007) in R 3.4.0 (R Development Core Team). Sampling groups were compared two‐by‐two. When comparing the sampling groups that were done repeatedly on the same colonies (Field May and Laboratory July), we included colony as a stand‐alone factor and tested its interaction with the sampling group. In the other comparisons which did not involve the repeated sampling of colonies (Field May vs. Field July and Laboratory July vs. Field July), colony was nested within sampling group. To keep the statistical models simple, we tested for each sampling group separately whether ant types (i.e., 0 days old, 5 days old, brood carers, foragers, and queens) differ in beta diversity metrics, using adonis with 999 permutations and colony always included as a blocking variable (strata).

We were interested whether there were differences between sampling groups in the variability of abdominal communities among and within colonies. Therefore, we compared pairwise between the sampling groups the Bray–Curtis distances (a) between members of different colonies and (b) between members of the same colony, with permutation tests estimated by Monte Carlo (999 permutations; R package perm). To control for multiple testing, the *p*‐values were subsequently adjusted with the Bonferroni correction.

To examine whether the sampling groups and ant types differ in alpha diversity, we calculated Shannon's diversity index for each sample with the alpha_diversity.py script of Qiime. The resulting values were compared with linear mixed effect (LME) models using the R package nlme. With the Field May samples, we tested for a relationship between bacterial abundance in the abdomen as estimated by qPCR and the Shannon's diversity index also with LME models. Colony was always included as a random effect in the LME models, and the residuals were visually checked for deviation from normality.

To obtain a measure of colony productivity that is controlled for colony size, we took for the Field May sampling group the total amount of brood items (eggs, larvae, and pupae) in a colony and divided it by the number of workers (per capita productivity). We used Spearman's rank correlations to test whether per capita colony productivity and colony size (number of workers) are correlated with the colony mean Shannon's diversity index or the colony mean bacterial abundance as estimated by the qPCRs targeting 16S copies. To test whether productivity is associated with the abdominal bacteria community composition of a colony, we averaged the OTU table over the individuals within each colony and used the R package GUniFrac (Chen et al., 2012) to compute weighted UniFrac distances for the table averaged for colonies. We chose to focus on weighted UniFrac distances because this method takes the phylogenetic relatedness of the bacteria into account and weighs the distances according to relative abundance, thus likely giving the most informative measure of abdominal community composition. We tested whether the colonies' abdominal communities are associated with per capita productivity and size with Mantel statistics based on Spearman's rank correlation as implemented in the R package vegan. An association between the eggs present in the nest when collected from the field (as a proxy for queen fertility) and queen weighted UniFrac distances was also examined with a Mantel test.

Any differences in abdominal communities between sampling groups and ant types according to the PERMANOVAs were further explored by identifying which OTUs or microbial families differed significantly in relative abundance between the groups of interest. The OTUs and microbial families that were present in <10% of the samples in all of the tests were removed from the normalized OTU table to prevent zero‐inflation and to reduce the amount of statistical tests. After removing the less abundant features, we conducted permutation tests with the fitLogNormal function of the metagenomeSeq R package (Paulson et al., 2013), using 100 permutations. The fitLogNormal function applies a log2 transformation to the data. To represent the differences in abundance of microbial families between the groups of interest, we used the log2 fold changes as provided by the fitLogNormal function (with “coef = 2”). The corresponding *p*‐values were adjusted with the “FDR” method. Taxonomic features with adjusted *p*‐values smaller than 0.05 were regarded as significantly different in abundance between the compared groups.

## 3 RESULTS

### 3.1 Community composition

**Figure 3:**
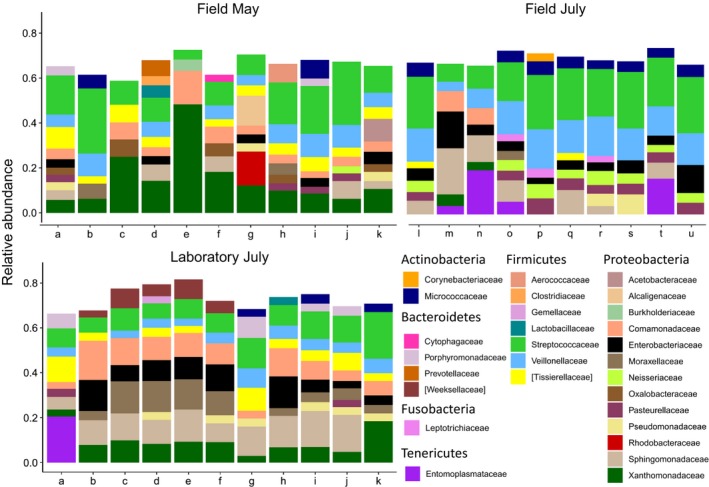
Relative abundances of bacterial families in the whole abdominal bacterial community of *Temnothorax* *nylanderi*, averaged over colony (the microbiome of 6, 12, and 9 workers was characterized per colony for the Field May, Laboratory July, and Field July group, respectively). In the legend, the bacterial families are ordered by phylum. For each colony, only the families are shown that amounted to at least 3% of the community composition. Colony means do not include the queen samples. Taxon names with square brackets are names proposed by the Greengenes database curators

**Figure 4:**
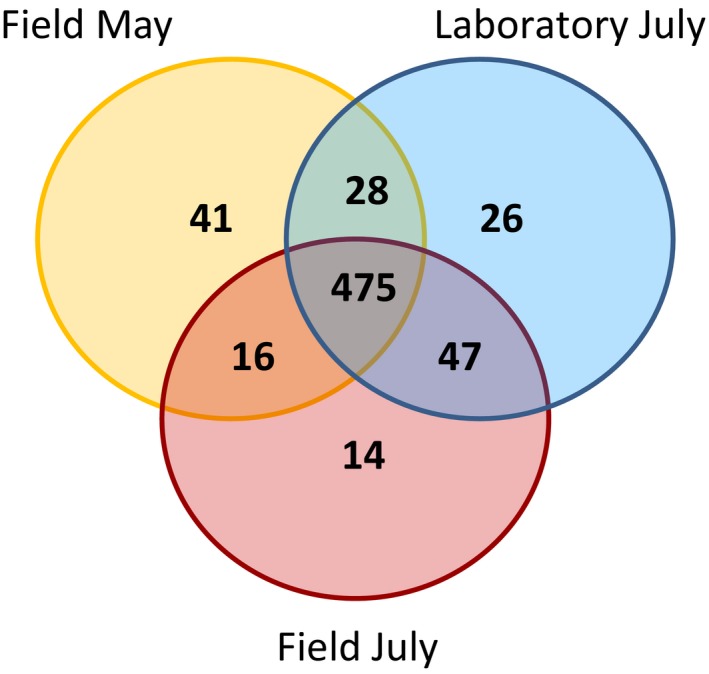
Venn diagram shows the number of OTUs shared between the three sampling groups

To visualize differences in abdominal bacterial community composition between colonies and between sampling groups, we averaged the relative abundances of the bacterial families for each colony and arranged them in stacked bars (Figure 3; for stacked bar plots of individual samples see Figures S3–S6): Multiple bacterial families repeatedly occurred in all three sampling groups, although their relative abundances varied considerable among the sampling groups (e.g., Moraxellaceae, Sphingomonadaceae, Xanthomonadaceae). The three sampling groups shared 475 of the 646 OTUs (Figure 4), suggesting that in general the bacterial communities of *T. nylanderi* ants are species rich and bacteria can be picked up by the environment resulting in different absence–presence patterns of OTUs among sampling groups. The stacked bars also showed that *T. nylanderi* colonies differ in their average bacterial community compositions even within sampling groups (Figure 3). For example, the Entomoplasmataceae varied strongly in relative abundance between colonies and bacteria of this family were not found in any of the ants of some colonies.

### 3.2 Beta diversity metrics

**Figure 5:**
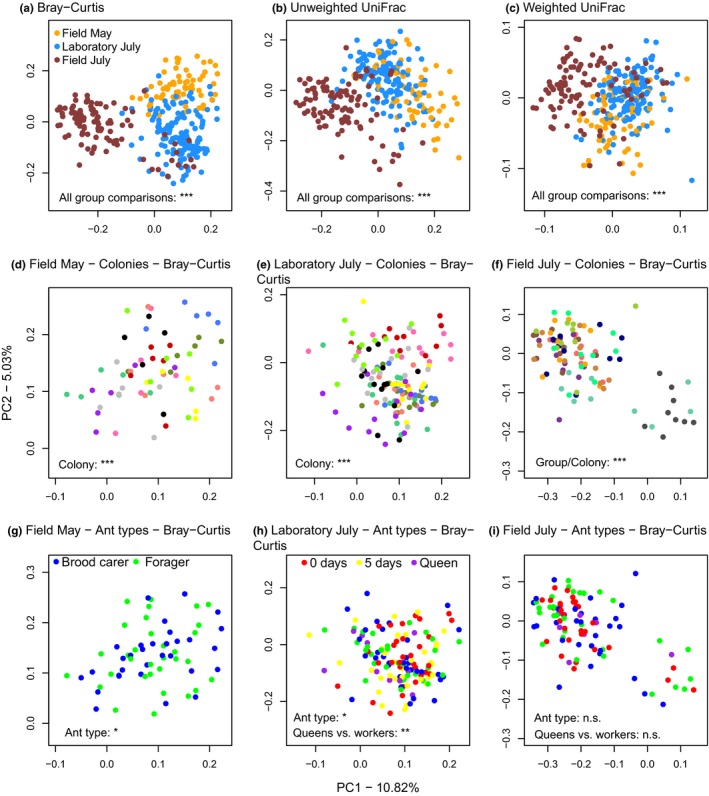
Principal Coordinate Analysis plots based on the beta diversity distances between *Temnothorax* *nylanderi* samples. The asterisks indicate the significance levels of factors tested in the PERMANOVAs, n.s. means “nonsignificant.” Individual ants significantly clustered by sampling group in *T. nylanderi*, for (a) Bray–Curtis distances, (b) unweighted UniFrac distances, and (c) weighted UniFrac distances. Additionally, the communities of members of the same colony significantly clustered (expressed in Bray–Curtis distance) for (d) Field May, (e) Laboratory July, and (f) Field July. Whether the sample originated from a brood carer or forager affected the Bray–Curtis distances significantly for the (g) Field May group. Also, ant type had a significant effect on the Bray–Curtis distances between the samples of the (h) Laboratory July group, which was due to a difference in Bray–Curtis distances between queens and workers for this sampling group. In contrast, we found no difference in Bray–Curtis distances between abdominal communities from different ant types for the (i) Field July sampling group

The abdominal bacteria communities clustered by sampling group in a principal coordinate (PC) analysis based on Bray–Curtis distances (Figure 5a–c). The clustering seemed less pronounced for the PC plots based on the beta diversity metrics that take phylogeny into account, that is, unweighted and weighted UniFrac distances (Figure 5b,c). However, the three sampling groups differed significantly from each other in all three beta diversity metrics (Table 1). Colony identity also significantly affected the similarity of the samples for all three beta diversity indexes (Table 1; Figure 5d–f): When the same colonies were sampled twice, once in May and again in July, samples taken from the same colony shared similarity (i.e., stand‐alone factor “Colony” is significant); however, the samples taken at different time points from the same colonies also differed significantly (significant interaction “Sampling group × colony”).

**Table 1:** PERMANOVA results for the comparisons of sampling groups

Comparison	Factor	Bray–Curtis dissimilarity			Unweighted UniFrac			Weighted UniFrac		
		Pseudo‐*F*	*R* ^2^	*p*	Pseudo‐*F*	*R* ^2^	*p*	Pseudo‐*F*	*R* ^2^	*p*
Field May versus Laboratory July	Group	10.92	0.05	**.001**	6.34	0.03	**.001**	12.32	0.05	**.001**
	Colony	1.94	0.10	**.001**	1.47	0.08	**.001**	1.78	0.09	**.001**
	Group × Colony	2.03	0.09	**.001**	1.62	0.07	**.001**	2.13	0.09	**.001**
Field May versus Field July	Group	26.19	0.13	**.001**	16.40	0.09	**.001**	26.11	0.13	**.001**
	Group/Colony	1.98	0.18	**.001**	1.47	0.15	**.001**	2.16	0.20	**.001**
Laboratory July versus Field July	Group	28.88	0.10	**.001**	13.51	0.05	**.001**	56.83	0.17	**.001**
	Group/Colony	2.13	0.16	**.001**	1.43	0.12	**.001**	2.86	0.18	**.001**

Note

The *p* values are based on 999 permutations. Significant *p*‐values are in bold. When comparing the sampling groups that were done repeatedly on the same colonies (Field May and Laboratory July), we included colony as a stand‐alone factor. In the comparisons that did not involve the repeated sampling of colonies (Field May vs. Field July and Laboratory July vs. Field July), colony was nested within sampling group.

Samples from the first sampling group (Field May) significantly clustered by ant type (Table 2 and Figure 5g), that is, foragers and brood carers differed in their abdominal microbiome, although the amount of variance explained was very low (*R* ^2^ = 0.02). Abdominal communities significantly differed with ant type in the Laboratory July group (Table 2 and Figure 5h). This was likely due to a significant difference in Bray–Curtis dissimilarity and unweighted UniFrac distances between the queen and worker samples for this group (Table 3). Indeed, when queens were excluded from the analysis, ant type was no longer significant (Bray–Curtis: Pseudo‐*F* = 0.87, *p* = .65; unweighted UniFrac: Pseudo‐*F* = 0.82, *p* = .92). Samples of the Field July group did not differentiate according to ant type (Table 2 and Figure 5i) and neither did queen and worker abdominal communities show any significant dissimilarity, although there was a trend for the Bray–Curtis distance (Table 3).

**Table 2:** PERMANOVA results for the comparisons of ant types within sampling groups

Group	Factor	Bray–Curtis dissimilarity			Unweighted UniFrac			Weighted UniFrac		
		Pseudo‐*F*	*R* ^2^	*p*	Pseudo‐*F*	*R* ^2^	*p*	Pseudo‐*F*	*R* ^2^	*p*
Field May	Ant type	1.14	0.02	**.03**	1.28	0.02	**.03**	1.32	0.02	.06
Laboratory July	Ant type	1.05	0.03	**.04**	1.03	0.03	.21	0.72	0.02	.58
Field July	Ant type	1.00	0.03	.17	1.04	0.03	.23	0.71	0.02	.88

Note

The *p* values are based on 999 permutations. Significant *p*‐values are in bold.

**Table 3:** PERMANOVA results for the comparisons of worker ants and queens within sampling groups Laboratory July and Field July

Group	Bray–Curtis dissimilarity			Unweighted UniFrac			Weighted UniFrac		
	Pseudo‐*F*	*R* ^2^	*p*	Pseudo‐*F*	*R* ^2^	*p*	Pseudo‐*F*	*R* ^2^	*p*
Laboratory July	1.54	0.01	**.003**	1.63	0.01	**.001**	0.32	0.00	.83
Field July	1.22	0.01	.06	1.14	0.01	.17	1.01	0.01	.29

Note

The *p*‐values are based on 999 permutations. The Laboratory July group contained 11 queens and 128 workers and the Field July group 8 queens and 89 workers. Significant *p*‐values are in bold.

**Figure 6:**
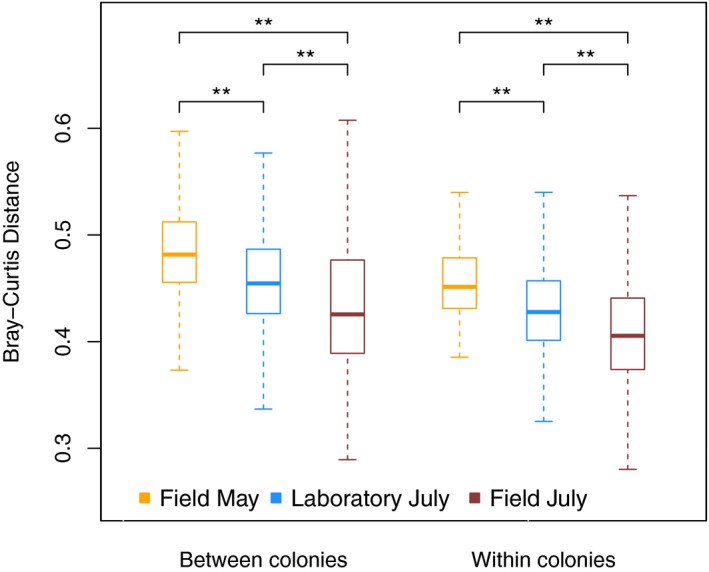
Bray–Curtis distances between microbial community profiles of members of different *Temnothorax nylanderi* colonies and between members of the same colony averaged for each sampling group. The horizontal bars of the boxplots indicate the medians and the boxes delimit the first and third quartile. The double asterisks indicate a significant difference between groups (*p* < .01)

The Bray–Curtis distances between the colonies of the Field May sampling group were larger than these distances within the two July sampling groups (Permutation test, both adj. *p* = .01; Figure 6). In turn, the Laboratory July colonies differed more from each other than the Field July colonies (Permutation test, adj. *p* = .01, Figure 6). The Bray–Curtis distances between members of the same colony were larger in the Field May group than in the Laboratory July group and the Field July group (Permutation test, adj. *p* = .01 and adj. *p* = .01, for both). The laboratory nestmates differed more from each other than the field nestmates collected in July (Permutation test, adj. *p* = .01).

### 3.3 Differences in relative abundances of bacterial families

**Figure 7:**
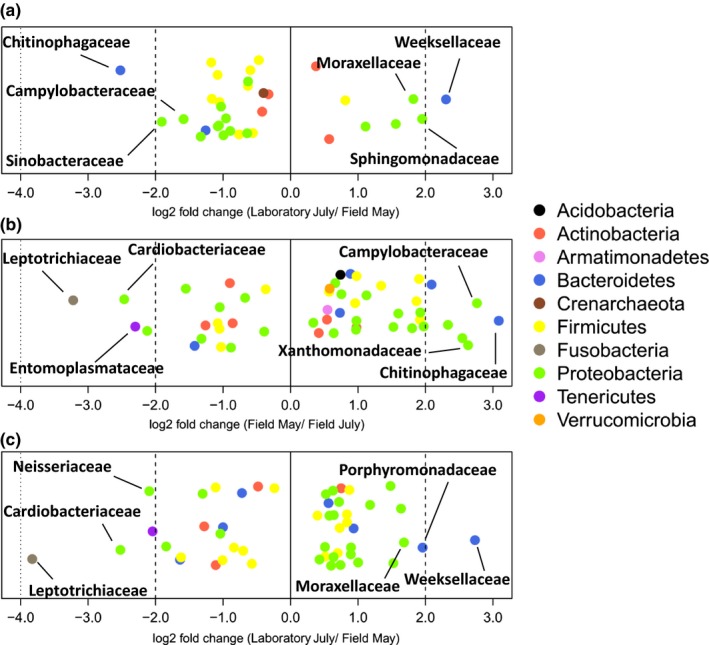
Log2 fold changes of the bacterial families that significantly differ (Permutation tests, adj. *p*‐value < 0.05) in abundance in pairwise comparisons of the *Temnothorax nylanderi* sampling groups: (a) Laboratory July relative to Field May, (b) Field May relative to Field July, and (c) Laboratory July relative to Field July. Each dot represents a bacterial or archaeal family; the three highest and lowest abundant families in comparison with the focal group are written on the plot. The families are colored by phylum

As many as 34 bacterial families significantly differed in relative abundance between the Field May and Laboratory July sampling group (Figure 7a; Table S4). The number of families which had a significantly higher or lower relative abundance between the Field May and the Field July group was with 52 even higher (Figure 7b), and also both July sampling groups showed a large difference with 50 families (Figure 7c). Because of the large number of families that differed in relative abundance between sampling groups, we mention mainly the families that had high log fold changes in relative abundance between sampling groups: The Field May and Laboratory July group both had significantly less Cardiobacteracea, Entomoplasmataceae, Neisseriaceae, and Leptotrichiaceae than the Field July group (Figure 7b,c and Table S4), which could mean that these families make up a larger part of the *T. nylanderi* abdominal community in summer, but only under natural conditions. Families that had a higher relative abundance in laboratory samples include the Sphingomonadaceae, Weeksellaceae, and the Moraxellaceae (Figure 7a,c). There were four families (Pasteurellaceae, Prevotellaceae, Streptococcaceae, and Veillonellaceae) that had lower relative abundance in the Laboratory July group than in both field sampling groups, suggesting these families are selected against by the laboratory environment (Table S3). The Field May group had the Campylobacteraceae, Chitinophagaceae, and Xanthomonadaceae in higher relative abundance than the two July sampling groups (Figure 7a,b), while it had the Enterobacteriaceae in notable lower relative abundance (Table S4). These changes in relative abundances between May and July could reflect seasonal fluctuations in the *T. nylanderi* abdominal microbiome.

**Figure 8:**
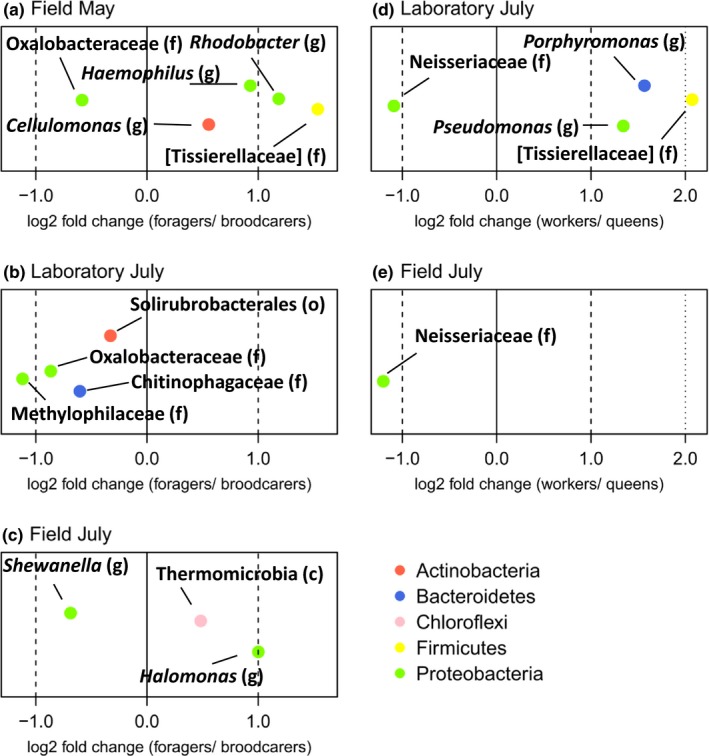
Log2 fold changes of the OTUs that significantly differed (adj. *p*‐value < 0.05) in their abundance between the abdominal communities of *Temnothorax nylanderi* foragers and brood carers for the three different sampling groups (a, b, and c) and between the communities of workers and queens for the two sampling groups in July (d and e). The lowest determinable taxonomic level of the OTU is indicated by the letter between brackets (c = class; o = order, f = family, and g = genus). A negative log fold change means that this OTU was more abundant in brood carers or queens, whichever is applicable. The OTUs are colored by phylum. Taxon names with square brackets are names proposed by the Greengenes database curators

We also determined the OTUs that significantly differed between brood carers and foragers for all sampling groups, because these were the only behavioral worker castes for which we found significant clustering by beta diversity indices. Although this effect was only significant for the Field May sampling group, analyzing all three groups allowed us to assess whether there were OTUs that were consistently different between brood carers and foragers. Only five OTUs significantly differed in abundance between brood carers and foragers collected from the field in May (Figure 8a and Table S5). The number of significantly different OTUs was only slightly lower for the Laboratory July and Field July group, four and three, respectively (Figure 8b,c and Table S5). There were no shared OTUs that differed significantly between brood carers and foragers for all three groups, but there was one OTU assigned to the family Oxalobacteraceae that was more abundant in brood carers for both the Field May and Laboratory July groups (Figure 8a,b and Table S5).

We found four OTUs that differed in abundance between queens and workers of the Laboratory July group (Figure 8d and Table S6). Only one OTU was significantly more abundant in queens than in workers sampled in the forest in July (Figure 8e and Table S6). Interestingly, this OTU, belonging to the family Neisseriaceae, was also more abundant in queens than in workers of the Laboratory July group (Figure 8d,e and Table S6).

### 3.4 Shannon's diversity index

**Figure 9:**
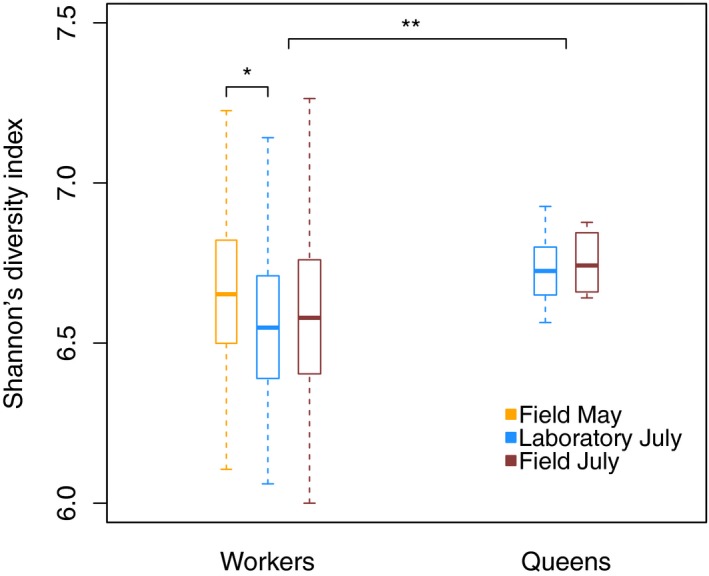
Shannon's diversity index grouped by *Temnothorax nylanderi* workers and queens for the sampling groups. The boxplots show medians, quartiles, and 5th and 95th percentiles

There were differences among the workers of the three sampling groups in the Shannon's diversity index (LME, *N* = 281, *F* = 3.14, *p* = .04): More specifically, field‐collected workers in May had a higher OTU richness than workers sampled from the same colonies in the laboratory in July (Tukey's post hoc, *z* = −2.50, *p* = .04; Figure 9). Workers from the two field sampling groups did not differ in Shannon's diversity (*z* = −1.10, *p* = .54) and neither did the laboratory workers differ from the workers collected from the field in July in OTU richness (*z* = 0.78, *p* = .54).

We also compared the Shannon's diversity index between the different ant types: In May, foragers and brood carers did not differ in Shannon's diversity (LME, *N* = 64, *F* = 1.93, *p* = .17). Also for the other two sampling groups, there were no differences in Shannon's diversity index depending on ant type (LME, Laboratory July, *N* = 139, *F* = 0.36, *p* = .55; Field July, *N* = 97, *F* = 0.83, *p* = .36). To test whether queen samples are more or less diverse than worker samples, we combined the data of both July sampling groups (i.e., the two sampling groups containing queens): Queens had a clearly higher Shannon's diversity index than workers (LME, *F* = 7.19, *p* = .01; Figure 9).

Bacterial abundance in the abdomen as estimated by the number of 16S copies via qPCR was unlinked to the Shannon's diversity index for the Field May samples (LME, *N* = 62, *F* = 0.75, *p* = .39; Figure S7). Moreover, the bacterial abundance, that is, the number of 16S copies, did not differ between Field May foragers and brood carers (LME, *N* = 62, *F* = 2.57, *p* = .12).

### 3.5 Colony productivity and queen fertility

**Figure 10:**
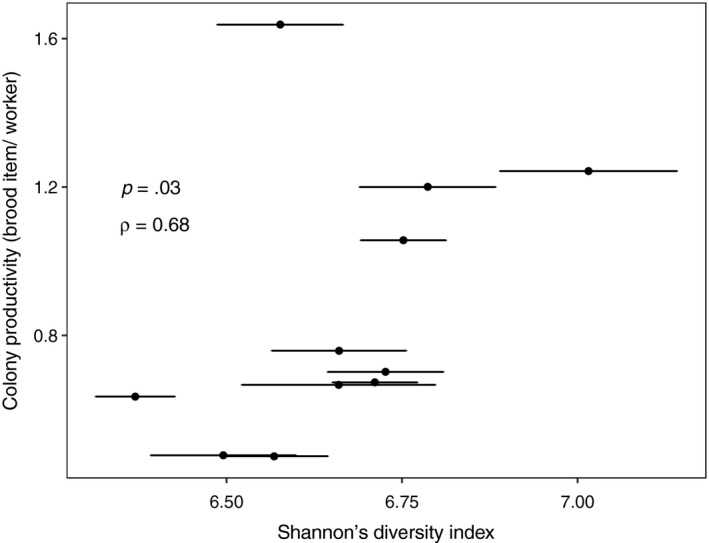
Correlation between *Temnothorax nylanderi* colony productivity expressed as the per capita colony productivity and a colony's mean microbiome Shannon's diversity index (Spearman rank correlation, results on plot). The error bars are standard errors

Shannon's diversity index was positively correlated with the per capita colony productivity (Spearman's rank correlation, *S* = 70, *ρ* = 0.68, *p* = .03; Figure 10), but not with colony size (Spearman's rank correlation, *S* = 286, *ρ* = −0.30, *p* = .37) for the colonies of the Field May sampling group. Neither per capita colony productivity nor colony size was associated with the weighted UniFrac distances between colonies (Mantel test based on Spearman's rank correlation, *r* = .13, *p* = .25 and *r *= −.08, *p* = .60, respectively). Also mean colony 16S copies were not correlated with per capita productivity and colony size (Spearman's rank correlation, *S* = 196, *ρ* = 0.11, *p* = .75 and *S* = 240, *ρ* = −0.09, *p* = .80, respectively).

However, queen fecundity, that is, the number of eggs in the nest upon collection, was linked to the weighted UniFrac distances of the queen abdominal communities (Mantel test based on Spearman's rank correlation, *r* = .56, *p* = .003), but not to the Shannon's diversity indices of the queen gut communities (Spearman's rank correlation, *S* = 216, *ρ* = 0.02, *p* = .96).

## 4 DISCUSSION

High‐throughput sequencing of the 16S rRNA gene allowed us to gain insights into the variation and consistency of bacterial communities in *T. nylanderi* ants and their association with the environment, sampling time, colony identity, and ant type. Relocating *T. nylanderi* colonies from the field to the laboratory changed the abdominal microbiomes, although our repeated collection of colonies from the field showed that sampling month can also partly determine microbiome composition in the abdomen, as both July sampling groups showed a reduced diversity of microbial communities compared with the colonies sampled in May. Abdominal microbiomes clustered according to colony identity in all sampling groups, an effect that was still detectable after colonies were housed in the laboratory for 2 months. There were no large differences between ant castes in abdominal microbiomes, although queens harbored slightly more diverse gut communities than workers. Interestingly, colonies with more diverse bacterial communities had more brood, and the number of eggs in the nest upon collection was associated with the queens' microbiome composition. Our study is the first to find associations between microbiome characteristics and colony productivity in social insects.

### 4.1 Gut bacterial communities of ants

Previous studies on ant gut microbiomes have observed associations of certain bacterial orders with the diet of the host, which suggests a symbiotic role of these broad taxonomic groups in ants (Anderson et al., 2012). *Temnothorax nylanderi* harbored bacterial taxa that were found to be associated with herbivorous, but also predatory and scavenging ant species (Anderson et al., 2012), possibly reflecting an opportunistic and wide dietary niche (Prebus, 2017). The total number of OTUs found in the *T. nylanderi* abdomen seems relatively large in comparison with other ants and insects in general. However, it is currently impossible to compare the diversity of gut communities quantitatively due to the methodological differences between studies. In a meta‐analysis of clone‐library‐based studies, Colman, Toolson, and Takacs‐Vesbach (2012) reported that different insect taxa harbor 11–103 OTUs (97% cutoff) per sample. Bacterial communities from bees are the least diverse with on average 11 OTUs per sample. This finding supports the idea that narrow dietary niches, such as the nectar and pollen diet of the honey bees, are associated with species‐poor gut communities (Martinson et al., 2011). Among three species of *Acromyrmex* leafcutter ants, a total of 180 OTUs were discovered (Sapountzis et al., 2015). This number seems comparatively low to the 646 we found for *T. nylanderi*. However, leafcutter ants also feed on a specialized diet, that is, their symbiotic fungus.

The composition of the *T. nylanderi* abdominal microbiome varies within and among colonies and sampling groups (Figures 3 and 4). As mentioned before, it is difficult to compare the community alpha diversity of *T. nylanderi* to other ant species due to differences in methodology between studies, and for the same reason, it is just as difficult to compare the intraspecific variability in community composition between ant species. However, the *T. nylanderi* abdominal microbiome seems to show a higher intraspecific variability in composition (Figure 3; Figures S3–S5) than the gut microbiomes of some other ant species, such as turtle ants (Sanders et al., 2017, 2014), Argentine ants (Hu et al., 2017), and Camponitini ants (Ramalho, Bueno, & Moreau, 2017; Sanders et al., 2017). Why some ant species have variable gut communities while others do not has not been investigated yet, but differences in community composition between ants of the same species have been linked to diet (Hu, Łukasik, Moreau, & Russell, 2014).

Many bacteria found in ant gut communities have no known or hypothesized function. However, in recent years progress has been made in identifying the role of some ant gut symbionts. The bacterium *Candidatus Tokpelaia hoelldoblerii*, a gut symbiont of the ponerine ant *Harpegnathos saltator*, has the genomic potential to synthesize vitamins and to recycle urea, a nitrogenous waste product in insect guts, into compounds that could be used by the ant host (Neuvonen et al., 2016). *Candidatus Tokpelaia hoelldoblerii* belongs to the family Bartonellaceae and the order of the Rhizobiales. Bacteria from the Bartonellaceae family are often found in ant guts (e.g., Anderson et al., 2012; Liberti et al., 2015; de Oliveira et al., 2016; Stoll, Gadau, Gross, & Feldhaar, 2007), and some have been hypothesized to recycle nitrogenous waste in favor of their ant hosts (Neuvonen et al., 2016; Russell et al., 2009). Surprisingly, *T. nylanderi* did not harbor any Bartonellaceae (Figure 3). The recycling of nitrogenous compounds is thought to be especially important for ant species feeding mostly on nitrogen‐poor plant saps: *Cephalotes* turtle ants, for example, obtain substantial amounts of essential amino acids with the help of bacterial lineages in the Burkholderiales, Opitutales, and Rhizobiales (Hu et al., 2018). The nitrogen that is upgraded by the bacteria presumably originates from nitrogenous waste in the ant gut, but also from bird droppings. We found members of the Burkholderiales in our study species (Figure 3), and they might also assist in nitrogen processing, as *T. nylanderi* workers also forage on vertebrate feces (Trabalon, Plateaux, Peru, Bagnéres, & Hartmann, 2000).

Previous studies on different ant species found both *Acinetobacter* (Liberti et al., 2015) and *Stenotrophomonas* (He, Wei, & Wheeler, 2014; Liberti et al., 2015) bacteria to be part of the gut microbiome. Interestingly, in the honey bee, *A. mellifera*, *Acinetobacter*, and *Stenotrophomonas* strains inhibit the growth of *Paenibacillus larvae*, a major honey bee pathogen (Evans & Armstrong, 2006). *Stenotrophomonas*, *Acinetobacter*, and other genera belonging to the same bacterial families (respectively, Xanthomonadaceae and Moraxellaceae, Figure 3) were also found to be regular members of *T. nylanderi* abdominal communities, making them interesting candidates for studies on the relationship between host health and gut symbionts in ants.

We detected in *T. nylanderi* another potentially interesting symbiont that belongs to the order Entomoplasmatales. Many different insect groups harbor these bacteria, which they have been observed to protect their hosts against parasitism (e.g., Jaenike, Unckless, Cockburn, Boelio, & Perlman, 2010), manipulate sex ratios (Hinrich et al., 2002), and cause disease (e.g., Meeus, Vercruysse, & Smagghe, 2012). In ants, Entomoplasmatales have been found in many species (Funaro et al., 2011; Ishak et al., 2011; Liberti et al., 2015; Łukasik et al., 2017; Oliveira et al., 2016; Sapountzis et al., 2015; Vieira, Ramalho, Martins, Martins, & Bueno, 2017). Although the symbiotic relationship between Entomoplasmatales bacteria and some ants is thought to be ancient (Funaro et al., 2011), their symbiotic role in ants is still unknown and worthy of further investigation. The OTU we found belonged to the genus *Entomoplasma*, and its abundance and prevalence differed considerably between colonies and sampling groups (Figure 3).

### 4.2 Environmental and seasonal variation in bacterial communities

The clustering of beta diversity distances by sampling group showed that the abdominal bacterial community of *T. nylanderi* is influenced by its environment (Figure 5a–c). Our experimental design, in which we sampled the same colonies twice, first in the field and 2 months later in the laboratory, complemented by samples from the field collected in the same time period, provides evidence that the differences between the Field May and the Laboratory July sampling group were not merely due to seasonal changes, but also because of laboratory conditions. A reduction in Shannon's diversity of termite gut communities was observed when laboratory colonies were repeatedly sampled over the course of 4 months (Benjamino & Graf, 2016). The *T. nylanderi* ants sampled in the laboratory in July did have a lower Shannon's diversity than the ones collected in the forest in May (Figure 9); however, they did not differ in alpha diversity from the Field July sampling group, which makes it difficult to rule out a general decrease in community diversity due to seasonality rather than to laboratory housing. Similarly, Koch, Cisarovsky, and Schmid‐Hempel (2012) repeatedly sampled the same bumble bee colonies and found a decrease in gut bacterial diversity with the progression of the season. By contrast, He et al. (2014) detected a greater bacterial diversity in laboratory‐raised than in field‐collected *Camponotus* ants; however, the samples were collected at different geographic locations and during different times of the year. Our results highlight the importance of controlling for seasonality when comparing the gut community characteristics of animals caught in the field and laboratory‐housed individuals.

The beta diversity distances between ants from the same and different colonies were larger for the Field May sampling group than for both other sampling groups (Figure 6). This suggests that laboratory conditions do not necessarily lead to a decrease in bacterial community diversity between and within colonies in *T. nylanderi* but that rather seasonality might be responsible for more similar communities among *T. nylanderi* ants. Seasonal cycles were described for mammals, including humans (e.g., Maurice et al., 2015; Smits et al., 2017), but there is little information on seasonality in bacterial communities in wild insect populations (but see Ferguson et al., 2018).

### 4.3 Between and within colony variation in bacterial communities

In *T. nylanderi*, nestmates have more similar abdominal communities than non‐nestmates (Table 1 and Figure 6). Moreover, individuals that were kept in the laboratory showed significant similarity with field‐collected individuals from the same colonies that were sampled 2 months before (Table 1). This suggests a role for the social group, genetics, or both in shaping the *T. nylanderi* abdominal microbiome. By composing a mixture of gut bacteria from 10 colonies of *Bombus terrestris*, and feeding this to germ‐free workers of those same colonies, Näpflin and Schmid‐Hempel (2018) showed that colony identity, and thus perhaps genetics, influences gut community composition in this bee. The underlying physiological mechanisms that link genetic background to gut community assembly are likely to be host immunity (e.g., Ryu et al., 2008) and gut physiology (e.g., McLoughlin, Schluter, Rakoff‐Nahoum, Smith, & Foster, 2016) and need to be investigated in social insects.

Our results also suggest differences in bacterial communities between individuals from the same colony, although these were typically smaller in magnitude compared with intercolony differences. The largest differences we found were between the physiologically most distinct individuals within a colony, the queens, and the workers. In *T. nylanderi*, queen communities had a higher Shannon's diversity than worker communities (Figure 9), while in termites and honey bees the opposite was the case (Kapheim et al., 2015; Poulsen et al., 2014). Apart from differing in alpha diversity, queens and workers of the Laboratory July group also weakly clustered according to their Bray–Curtis and unweighted UniFrac distances, but not according to their weighted UniFrac distances (Table 3): This indicates that queens and workers harbored some different bacteria, which did not share close phylogenetic relationships, but that these bacteria in general were not highly abundant in the ants. A possible explanation could be that upon relocation to the laboratory, workers were quicker to adopt new bacterial strains than queens. In line with this, samples of the Field July group did not cluster by caste (Table 3).

In both July sampling groups, queens had relatively higher abundances of Neisseriaceae bacteria than workers (Figure 8), suggesting a caste‐related symbiotic role for these bacteria. An online BLAST search of the corresponding OTU sequence against the 16S rRNA (Bacteria and Archaea) database of the NCBI gave as the closest match (93.0% identity) a sequence belonging to *Snodgrassela alvi* (NR_121735.2), a dominant member of honey bee (*Apis* spp.) and bumble bee (*Bombus* spp.) gut communities (Kwong, Engel, Koch, & Moran, 2014). In the gut of the European honey bee (*A. mellifera*), *S. alvi* is thought to utilize carboxylic acids as an energy source (Kešnerová et al., 2017), but any positive or negative effects of this bacterium on its host have so far not been described.

In addition, we found a difference between brood carers and foragers, but only for the Field May sampling group (Table 2). It is difficult to hypothesize why only the brood carers and foragers of the Field May group differed, but it might be due to seasonal changes in age of the worker castes. Interestingly, there was one OTU assigned to the family Oxalobacteraceae that was more abundant in brood carers than in foragers in both the Field May and Laboratory July groups. This family belongs to the Burkholderiales, which, as mentioned before, have been shown to recycle nitrogenous waste in turtle ants (Hu et al., 2018). Thus, metabolic differences between *T. nylanderi* brood carers and foragers (Kohlmeier et al., 2019) might explain the difference in relative abundance of Oxalobacteraceae.

Although their age and physiology must greatly differ from workers such as foragers, *T. nylanderi* callows did not have less diverse communities than older workers. A similar result was found for fire ants (Ishak et al., 2011) and leafcutter ants (Zhukova et al., 2017). Hongoh et al. (2006) observed greater similarities between gut communities of same‐aged termites than between colony members. However, workers of this termite species have different diets depending on their age (Hongoh et al., 2006): Young workers ingest plant material, while older ones mainly eat from the fungus they rear. In *T. nylanderi* and many other ants, changes in diet with age are likely to be more subtle. As our study only looked at the relative abundance of bacteria, we cannot rule out that newly eclosed workers greatly differ from older workers in the absolute number of bacteria in their abdomens. Additionally, the time window in which *T. nylanderi* callows acquire their bacterial symbionts might be very shortly after eclosion, and by selecting ants with light cuticles, we might have mainly sampled individuals that were already colonized by bacteria. To more precisely determine when the gut communities of *T. nylanderi* workers are established, future studies could include larvae and pupae.

### 4.4 Abdominal microbiome characteristics and colony phenotype

An important role of microbial symbionts for host fitness has been found for a taxonomically diverse group of insects (e.g., Coon, Brown, & Strand, 2016; Reyes et al., 2019; Schwab, Riggs, Newton, & Moczek, 2016). Thus, we tested for associations between abdominal microbial community composition and proxies for colony fitness such as brood quantity and colony size. For *T. nylanderi*, we found that a colony's productivity was related to the mean Shannon's diversity index of its abdominal community (Figure 10), suggesting that a more diverse microbiome could benefit worker rearing. A diverse gut community could contribute to colony health and nutrition in various ways: Certain gut bacterial community assemblies could prevent the colonization of pathogens (e.g., Dillon, Vennard, Buckling, & Charnley, 2005), and bacterial species can have complementary pathways to degrade or recycle certain compounds (e.g., Hu et al., 2018). As there was no relationship between bacterial abundance and Shannon's diversity index, we do not think that our measure of bacterial community diversity is confounded by differences in abdominal bacterial abundance between colonies. However, factors such as colony age (Koch et al., 2012), foraging behavior (Leitão‐Gonçalves et al., 2017; Wong et al., 2017), and genetics (Näpflin & Schmid‐Hempel, 2018) might drive an association between colony productivity and the diversity of the worker abdomen community. Experiments manipulating the microbiome could establish whether there is a causal link between community diversity and colony productivity in *T. nylanderi*. Studies that have radically disrupted bacterial communities in both social and nonsocial insects by administering antibiotics have found strong fitness effects (e.g., Jaffé et al., 2001; Nogge & Gerresheim, 1982; Rosengaus, Zecher, Schultheis, Brucker, & Bordenstein, 2011), though ideally controlled inoculations with bacterial strains should be performed to test for the effects of the community composition on the host (e.g., Kešnerová et al., 2017; Schwab et al., 2016).

Additionally, we found for *T. nylanderi* that the number of eggs in field nests was linked to the clustering of queen microbiomes according to the weighted UniFrac distances. If we assume that the number of eggs in a nest upon collection reflects a queen's fecundity, then our results indicate that a queen's abdominal microbiome might affect her egg production and ultimately colony fitness. This is in line with other studies on insects, which found that bacterial gut residents can influence egg production (e.g., Ben‐Yosef, Pasternak, Jurkevitch, & Yuval, 2014; Berasategui et al., 2017; Rosengaus et al., 2011). However, our observation could be confounded by factors such as diet, health, and genetics. Therefore, targeted experiments are necessary that more directly measure queen fertility (egg laying rate) in individuals of known sizes, age, and on a similar diet. Which bacterial taxonomic groups are correlated with the number of eggs in the nest is difficult to determine because of the many different bacterial taxa, especially for such a highly diverse community as *T. nylanderi's* (Figure S8).

### 4.5 Concluding remarks

We found that the abdominal bacterial community of the omnivorous ant *T. nylanderi* is highly diverse, which might reflect the generalist and opportunistic dietary niche of this species. Another interesting aspect of our study is the discovery of colony‐specific abdominal microbiomes, which remain at least partly intact after transfer to laboratory conditions. This suggests a role of host genetics, the social environment, or both in shaping the *T. nylanderi* abdominal community. Future studies could explore which genetic or behavioral mechanisms generate colony‐specific microbiomes in ants. On top of that, we observed that colony microbial community diversity is positively correlated with productivity in a social insect, and as colony productivity is a potential determinant of colony fitness, this finding warrants further investigation.

## CONFLICT OF INTEREST

None declared.

## AUTHOR CONTRIBUTIONS

All authors contributed to the design of the study. F.H.I.D.S. performed the research and analyzed the data. All authors helped to interpret the data and worked on the manuscript.

## Data Availability

All 16S rRNA sequences have been deposited in the Sequence Read Archive (SRA) of the NCBI with links to BioProject PRJNA574251.
