# Supplementary material for: Abdominal microbial communities in ants depend on colony membership rather than caste and are linked to colony productivity
Source: Ecol Evol. 2019 Nov 14;9(23):13450–67. doi: 10.1002/ece3.5801 (PMC6912891; doi:10.1002/ece3.5801)

**Supplementary Figure 1.** Rarefaction curve for the observed Operational Taxonomic Units (OTUs). The lines are drawn through the means which were calculated from the rarefaction results of a hundred iterations starting at a hundred and stopping at 1500 sequences randomly drawn from each sample, in increments of a hundred. The error bars represent the standard errors of the means.


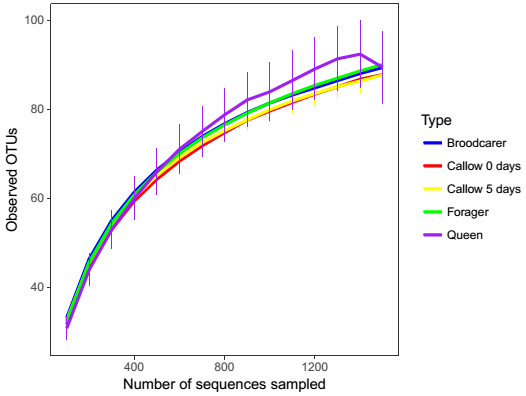


**Supplementary Figure 2.** In an ordination analysis samples do not cluster by sequencing depth. The points represent individual samples, those with relatively low amount of sequences are coloured red (800 to 1200 sequences) and blue (1201 to 1600 sequences), while the samples with more than 1600 sequences are shown in grey. The Principal Coordinates (PCs) were calculated based on (a) Bray-Curtis Distances, (b) Unweighted UniFrac Distances and (C) Weighted UniFrac Distances.


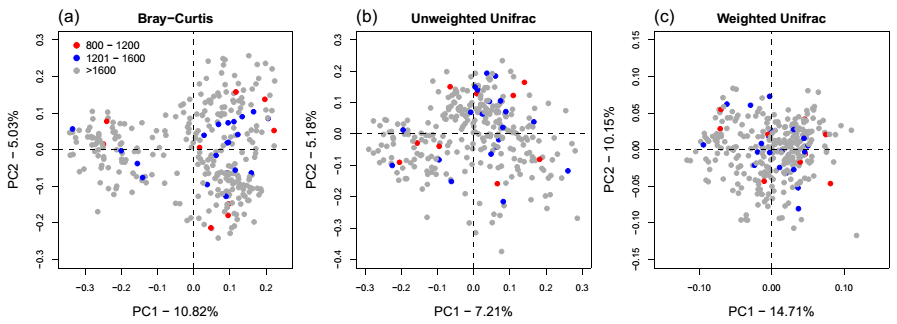


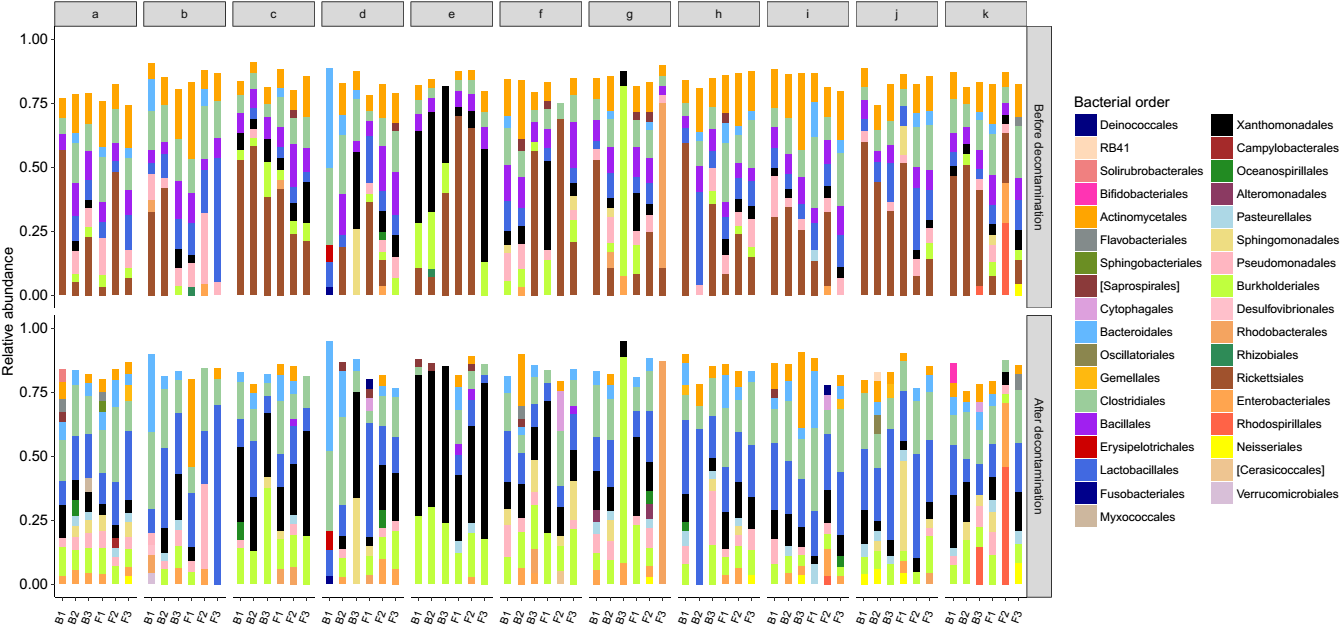


**Supplementary Figure 3.** The abundances of bacterial orders relative to the whole abdominal bacterial community for the individual samples of sampling group Field May, sorted by colony and ant type (B=broodcarer; F=forager). Only the orders are shown that contributed at least 3% to the community composition of one sample. The upper panel displays the community composition for each sample before OTUs were removed that belonged to contaminants and *Wolbachia* (order Rickettsiales). This figure also includes the samples which were excluded from the data analysis due to low sequence amounts.

**
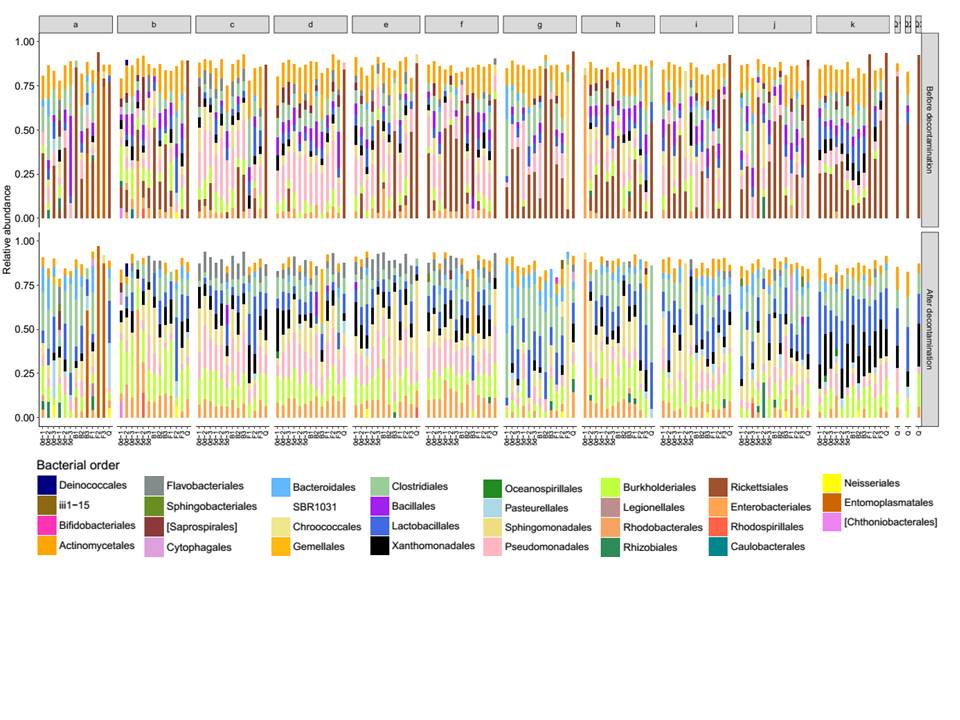
**

**Supplementary Figure 4.** The abundances of bacterial orders relative to the whole abdominal bacterial community for the individual samples of sampling group Laboratory July, sorted by colony and ant type (0d=0 days; 5d=5 days; B=broodcarer; F=forager; Q=queen). Only the orders are shown that contributed at least 3% to the community composition of one sample. The upper panel displays the community composition for each sample before OTUs were removed that belonged to contaminants and *Wolbachia* (order Rickettsiales). This figure also includes the samples which were excluded from the data analysis due to low sequence amounts.


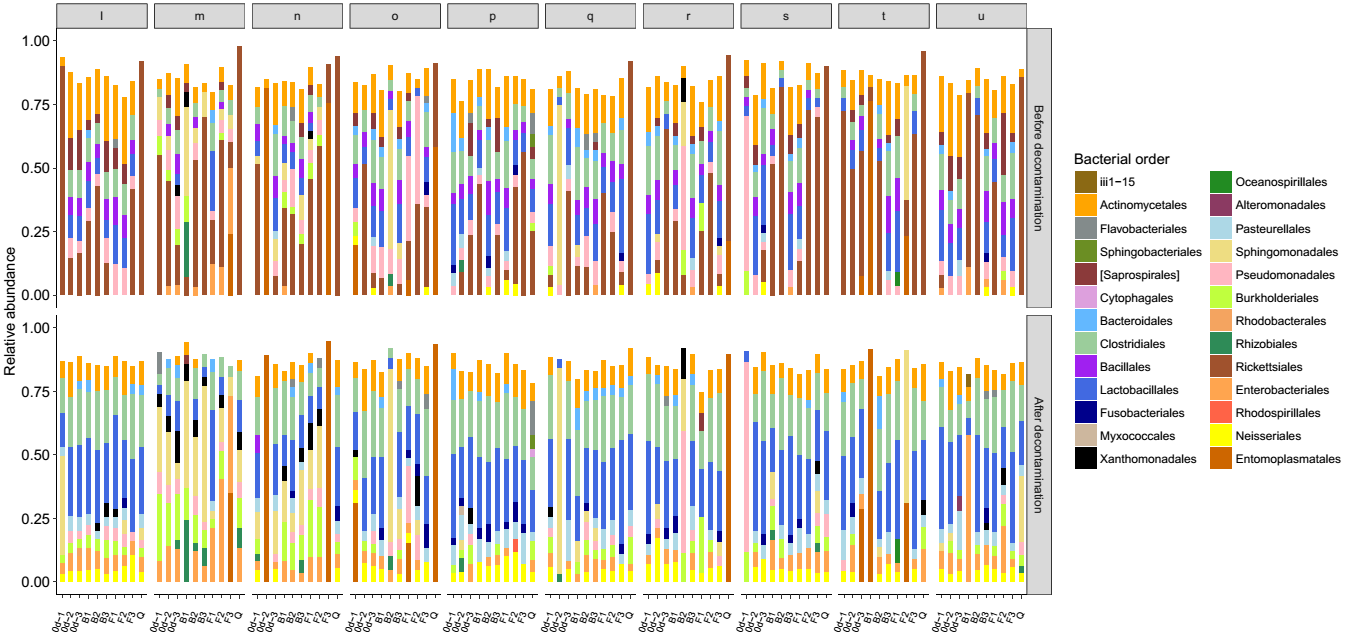


**Supplementary Figure 5.** The abundances of bacterial orders relative to the whole abdominal bacterial community for the individual samples of sampling group Field July, sorted by colony and ant type (0d=0 days; B=broodcarer; F=forager; Q=queen). Only the orders are shown that contributed at least 3% to the community composition of one sample. The upper panel displays the community composition for each sample before OTUs were removed that belonged to contaminants and *Wolbachia* (order Rickettsiales). This figure also includes the samples which were excluded from the data analysis due to low sequence amounts.

**Supplementary Figure 6.** The abundances of bacterial orders relative to the whole community for the control samples. A “(c)” behind a taxonomic group listed in the legend indicates that the order could not be determined and that instead the bacterial class is given. Only the orders are shown that contributed at least 3% to the community composition of one sample. The control samples were as follows: C1=0.1%SDS and distilled H_2_O; C2=distilled H_2_O in which dissection tools were dipped; C3=0.1%SDS and distilled H_2_O; C4=0.1%SDS and distilled H_2_O; C5=distilled H_2_O in which dissection tools were dipped after they were rubbed against dissection surface; C6=cricket leg (ant food); C7=honey (ant food) dissolved in distilled H_2_O; C8=lab tap water; C9=swab of plaster nest; C10=swab of plaster nest; C11=cricket leg taken from nest.


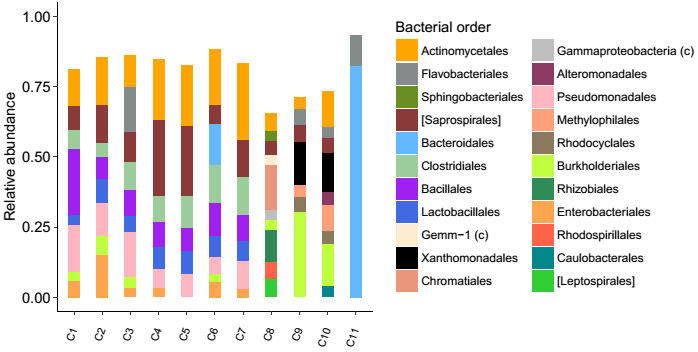


**Supplementary Figure 7.** The total amount of 16S rRNA copies in a sample plotted against Shannon’s diversity index for the individual samples of the Field May sampling group. We found no relationship between the amount of 16S copies in the samples and Shannon’s diversity index (LME, N=62, F=0.75, p=0.39).

**
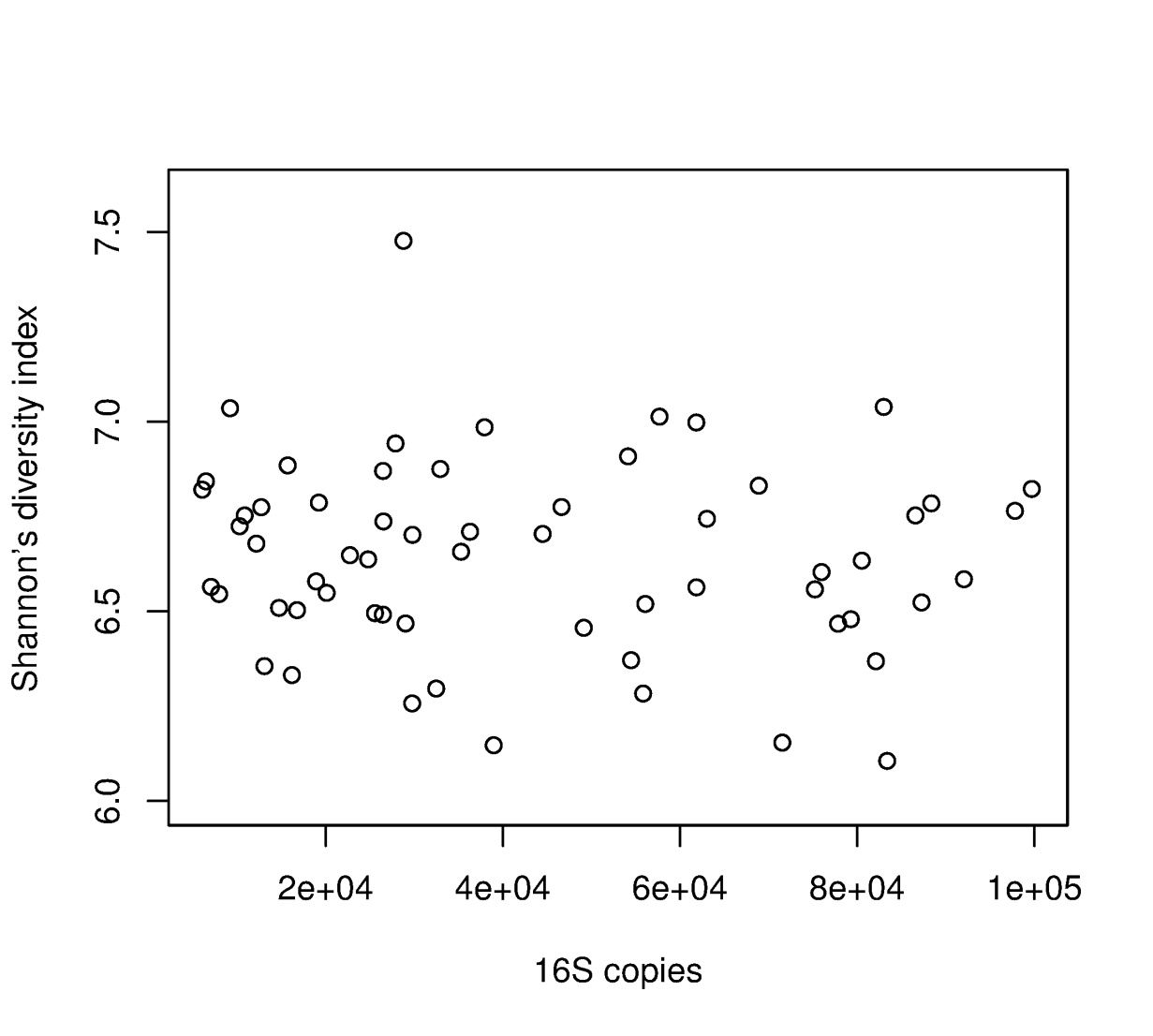
**

**Supplementary Figure 8.** The stacked bars show the relative abundances of bacterial families to the whole queen abdominal bacterial communities, in order of the amount of eggs present in the nest upon collection. The order of the bacterial families in the legend is the same as the order with which they are shown in the stacked bars from top to bottom. For each queen only the families are shown that contributed equal to or more than 2% to the community composition.


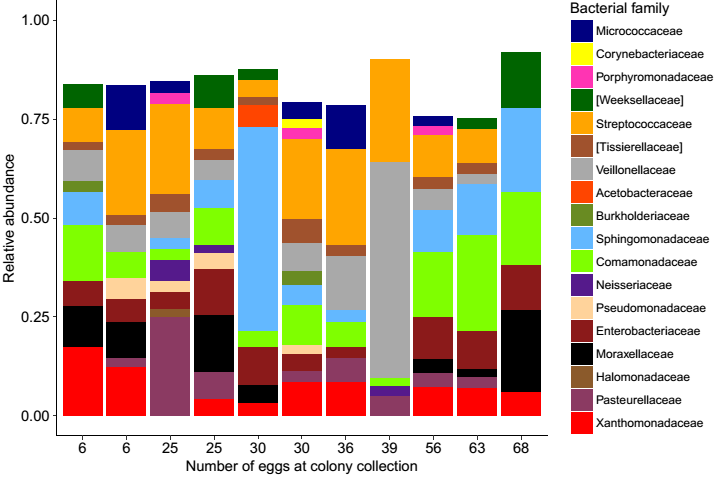

Supplement: Supplementary file 1 [file ECE3-9-13450-s001.docx]
